# Trifurcation of the Right Coronary Artery With Myocardial Bridging of the Posterior Interventricular Artery and the Absence of the Left Marginal Artery: A Report of a Cadaveric Case

**DOI:** 10.7759/cureus.101266

**Published:** 2026-01-10

**Authors:** Juma Mwalimu, Justus A Kamara, Ivony I Kamala, Dalila Mwindadi, Mukasa Mohammed, Kalkidan Mekoya, Shabbir Adamjee, Gibonce Mwakisambwe, Sugra S Fazal, Lisa D Kyamarisi

**Affiliations:** 1 Anatomy, St. Joseph University in Tanzania, Dar es Salaam, TZA; 2 Anatomy, Sri Ramachandra Institute of Higher Education and Research, Chennai, IND; 3 Medicine, Saifee Hospital Zanzibar Ltd., Dar es Salaam, TZA; 4 Medicine, Aenon Health Care, Dar es Salaam, TZA; 5 Public Health, Aenon Health Care, Dar es Salaam, TZA; 6 Internal Medicine, Saifee Hospital Zanzibar Ltd., Dar es Salaam, TZA; 7 Surgery, Saifee Hospital Zanzibar Ltd., Dar es Salaam, TZA; 8 Critical Care Medicine, Saifee Hospital Zanzibar Ltd., Dar es Salaam, TZA; 9 Internal Medicine, Kairuki Hospital, Dar es Salaam, TZA

**Keywords:** cadaver case report, coronary artery variations, coronary collateral circulation, myocardial bridging, right coronary artery trifurcation

## Abstract

Anatomical variations of the coronary arteries are common; however, the simultaneous occurrence of branching and course anomalies affecting both the right and left coronary systems is rare. This report describes a unique triad of coronary variations observed during the routine dissection of a 50-year-old formalin-fixed male cadaver. The right coronary artery trifurcated in the posterior atrioventricular groove into two distinct posterior interventricular arteries and a continuing atrial branch, with the dominant posterior interventricular artery exhibiting a 5-mm myocardial bridge as it traversed the ventricular musculature before resurfacing. On the left side, the marginal artery branches were absent, and the lateral wall of the left ventricle was supplied by compensatory branches arising from the left circumflex artery and the right coronary artery. While trifurcation patterns are more frequently documented on the left side, trifurcation of the right coronary artery and myocardial bridging of the posterior interventricular artery are considerably less common. These variations have the potential to alter coronary hemodynamics and pose challenges during surgical or interventional procedures, while the observed compensatory collateral supply underscores the adaptive capacity of coronary perfusion. Precise awareness of such rare combined coronary anomalies is essential for cardiothoracic surgeons and interventional practitioners to prevent iatrogenic injury and to accurately interpret atypical patterns of myocardial ischemia.

## Introduction

The standard anatomical configuration of the coronary circulation typically involves two main ostia arising from the aortic sinuses, giving rise to the left coronary artery and the right coronary artery [1]. Normally, the left coronary artery bifurcates into the left anterior descending artery and the left circumflex artery, with the left anterior descending artery providing several diagonal branches that supply the anterolateral wall of the left ventricle [1,2]. The right coronary artery usually courses within the coronary sulcus and, in a right-dominant system, gives rise to the posterior interventricular artery, also referred to as the posterior descending artery, which typically follows an epicardial course along the posterior interventricular groove [1,3].

Despite this conventional pattern, the coronary arterial tree demonstrates considerable morphological variability. Branching variations such as trifurcation are relatively well documented within the left coronary system; however, trifurcation of the right coronary artery is an infrequent anatomical finding [4,5]. Additionally, a complete absence of marginal branches is uncommon and implies a compensatory redistribution of blood supply through adjacent vessels, most frequently the left circumflex artery or, less commonly, the right coronary artery [1,2]. Another notable coronary variation is myocardial bridging, a condition in which a segment of a coronary artery takes an intramyocardial rather than an epicardial course [6,7].

The embryological basis of such anomalies is rooted in the complex development of the coronary vasculature. During early cardiac morphogenesis, the subepicardial vascular plexus undergoes extensive remodeling before establishing connections with the aortic sinuses. Disruptions or persistence of primitive vascular channels during this process may result in atypical branching patterns or absence of specific vessels, such as diagonal arteries [1,8]. Myocardial bridging is believed to arise during myocardial compaction, when developing cardiac muscle overgrows a previously superficial coronary segment, resulting in an intramyocardial course [7].

From a clinical perspective, recognition of these variations is essential. Although many coronary anomalies remain asymptomatic, a trifurcating right coronary artery may complicate coronary angiography or interventional procedures and alter expected ischemic patterns [4,5]. Similarly, myocardial bridging has been associated with systolic compression of the involved artery and may contribute to angina, arrhythmias, or myocardial ischemia in the absence of atherosclerotic disease [6,7]. Awareness of such anatomical deviations is therefore critical for cardiothoracic surgeons and interventional cardiologists to prevent diagnostic errors and iatrogenic injury [1].

## Case presentation

During routine anatomical dissection of a formalin-fixed South Indian male cadaver (approximately 50 years of age), several notable variations in the branching and course of the coronary arteries were identified. The cause of death was unrelated to the findings described herein.

**Figure 1:**
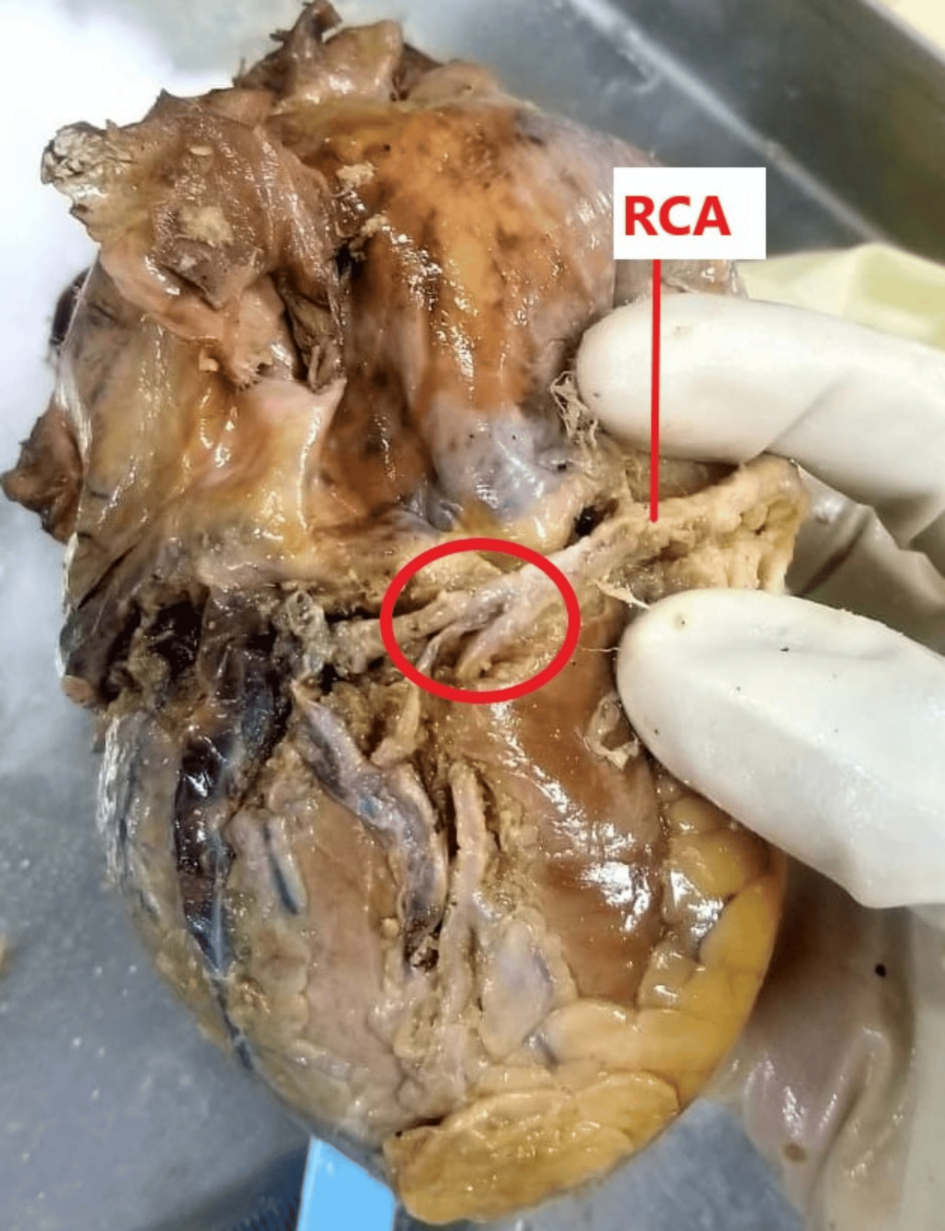
Trifurcation of the right coronary artery (RCA). The diaphragmatic surface of the heart is shown, exposing the posterior atrioventricular groove. The RCA demonstrates trifurcation at the level of the crux of the heart (red circle).

The right coronary artery, while coursing within the right posterior atrioventricular groove, exhibited a rare terminal trifurcation. This trifurcation gave rise to two distinct posterior interventricular arteries, with the parent vessel subsequently continuing its course at the level of the crux of the heart (Figure 1).

**Figure 2:**
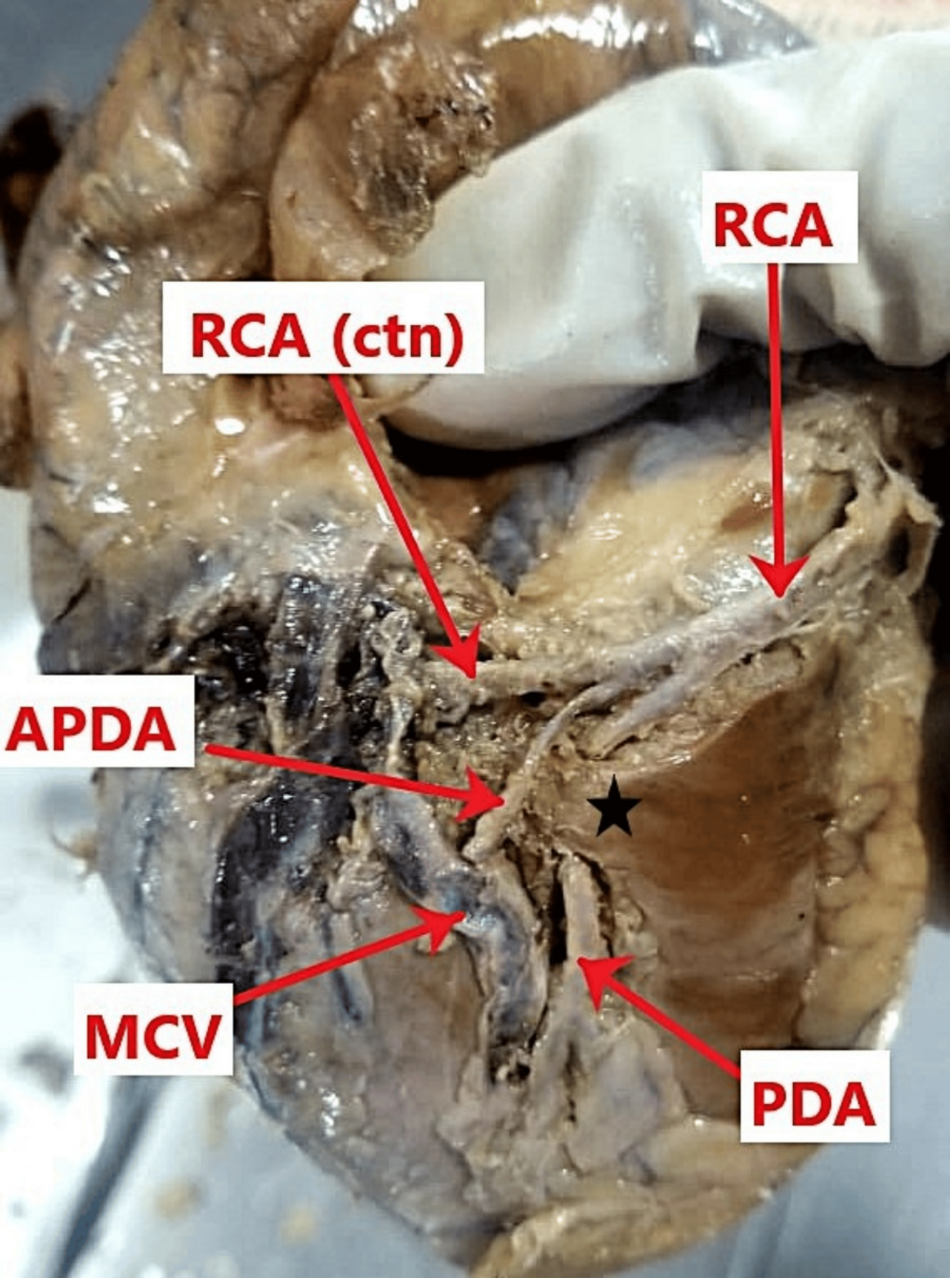
Course of the right coronary artery (RCA) branches. The diaphragmatic surface of the heart shows the dominant posterior interventricular artery (posterior descending artery, PDA) taking an intramyocardial course consistent with myocardial bridging (black star). The accessory posterior descending artery (APDA) accompanies the middle cardiac vein (MCV) and then courses within the posterior interventricular groove alongside the dominant PDA.

The dominant posterior interventricular artery branch immediately exhibited an intramyocardial course, creating a myocardial bridge. Specifically, the vessel pierced the ventricular musculature and traveled intramyocardially for a distance of approximately 5 mm before emerging to resume its epicardial course within the posterior interventricular groove. An accessory posterior interventricular artery branch was also observed, which followed a superficial course alongside the middle cardiac vein within the posterior interventricular groove (Figure 2).

**Figure 3:**
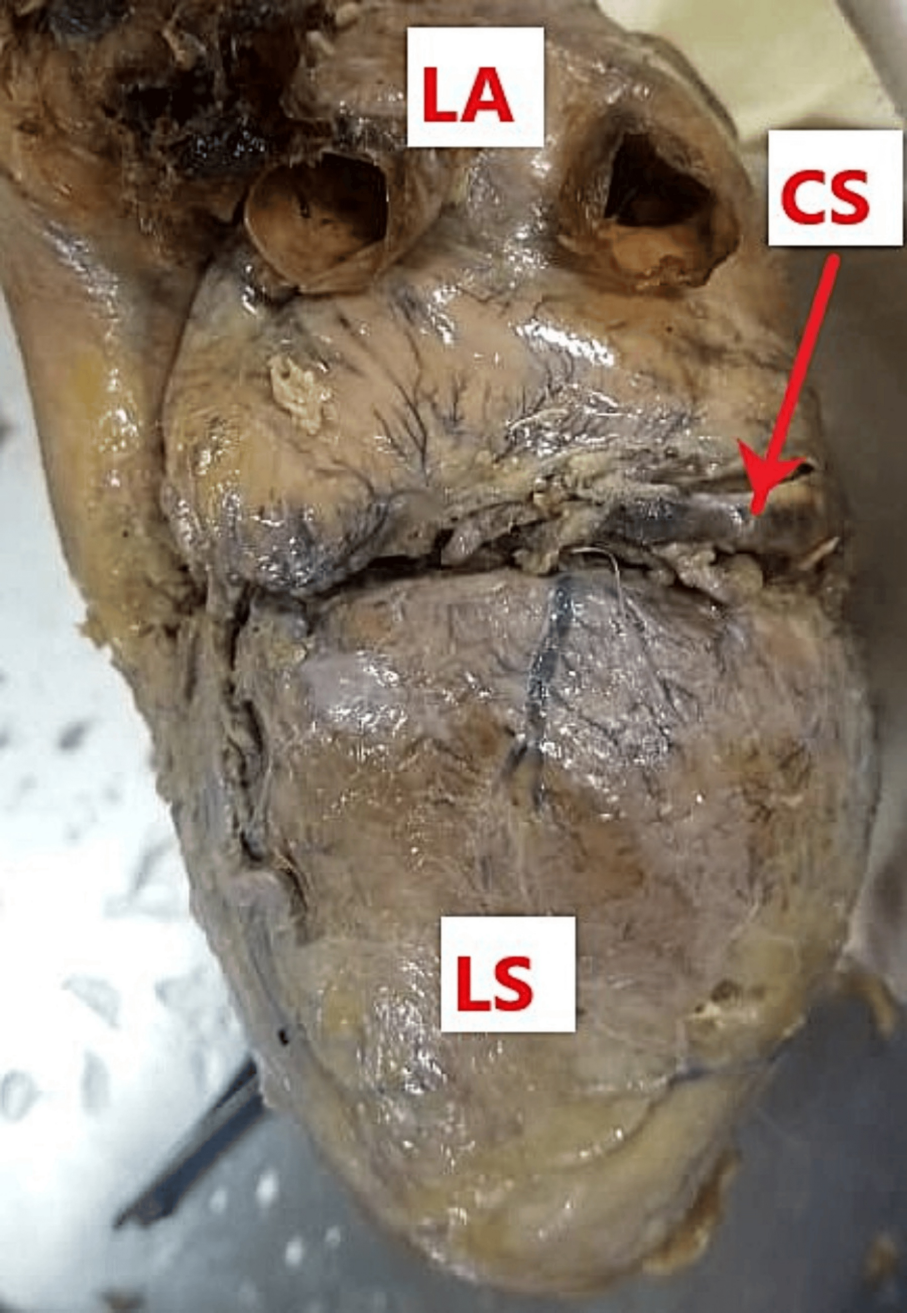
Left surface of the heart. The left marginal artery is absent on the left surface (LS) of the heart. LA: left atrium; CS: coronary sinus

Upon inspection of the left surface of the heart, there was a complete absence of the left marginal artery. The left surface of the heart was instead perfused by a collateral network of smaller vessels. These compensatory branches originated from the left circumflex artery, the left anterior descending artery, and the terminal branches of the right circumflex artery (Figure 3).

The remaining surfaces of the heart, including the right atrium and the anterior surface of the right ventricle, displayed standard vascularization. The proximal origins of both the left and right coronary arteries from the aortic sinuses were normal, and the venous drainage showed normal anatomy.

The study was conducted in accordance with the ethical principles of the Declaration of Helsinki [9], using cadaveric specimens sourced from the institution’s anatomy department under approved protocols for educational and research purposes.

## Discussion

The present case demonstrates a rare and complex constellation of coronary artery variations occurring within a single specimen. The findings include a terminal trifurcation of the right coronary artery giving rise to two distinct posterior interventricular arteries, the presence of a myocardial bridge involving the dominant posterior interventricular artery, and the complete absence of left marginal artery branches. The coexistence of both branching and course anomalies represents a highly unusual coronary morphological pattern that deviates substantially from typical anatomy [4-6].

Anatomical studies have shown that coronary variations are more frequently encountered within the left coronary system, particularly involving the left main coronary artery. While trifurcation of the left coronary artery has been reported with variable prevalence, trifurcation of the right coronary artery remains exceedingly rare and is only sporadically described in cadaveric and angiographic studies [4,5]. Similarly, myocardial bridging most commonly affects the left anterior descending artery, whereas involvement of the posterior interventricular artery is uncommon and reported at a substantially lower frequency [3,7]. The complete absence of the left marginal artery is also infrequent, given its usual contribution to perfusion of the lateral left ventricular myocardium [1,2].

Duplication of the posterior interventricular artery has been documented in rare cases; however, in most reports, these vessels arise independently rather than from a true trifurcation at the crux of the heart, as observed in the present case [4,5]. In addition, while previous studies have described compensatory enlargement of the left anterior descending artery or the left circumflex artery in the absence of diagonal branches, the current specimen demonstrated a combined collateral supply from both the left circumflex artery and terminal branches of the right coronary artery, indicating a high degree of adaptability in coronary perfusion [1]. The myocardial bridge identified in the dominant posterior interventricular artery was short but intramyocardial, consistent with deeper forms of bridging that may carry greater clinical relevance because of the potential for systolic compression [7].

Persistence of multiple primitive vessels during formation of the posterior coronary circulation may explain the trifurcation of the right coronary artery, whereas failure of selective angiogenic sprouting may account for the absence of diagonal branches [1,8]. The intramyocardial course of the posterior interventricular artery likely developed during myocardial compaction, when overlying cardiac muscle enveloped the vessel rather than allowing it to remain within the epicardial groove [7].

From a clinical standpoint, these variations have important implications. Myocardial bridging involving the posterior interventricular artery may predispose affected individuals to myocardial ischemia, arrhythmias, or atypical chest pain due to dynamic systolic compression [6,7]. Furthermore, the presence of a trifurcating right coronary artery combined with the absence of marginal branches significantly alters the expected coronary anatomical roadmap, which is critical during coronary angiography, coronary artery bypass grafting, and other interventional procedures [1,4,5]. Failure to recognize such anomalies may lead to misinterpretation of imaging findings or inadvertent vascular injury.

## Conclusions

In conclusion, this case provides a rare anatomical documentation of a trifurcating right coronary artery associated with a dual posterior interventricular artery system and an intramyocardial arterial course. The simultaneous absence of left marginal branches underscores the heart’s capacity for compensatory collateralization through the left circumflex artery and right coronary artery systems. This unique triad of anomalies highlights the necessity for meticulous anatomical awareness and precise imaging during invasive cardiac procedures to prevent iatrogenic complications. Ultimately, this report contributes to the broader understanding of coronary morphological diversity and its potential implications for clinical outcomes.
